# Supplementary material for: Mechanisms governing the pioneering and redistribution capabilities of the non-classical pioneer PU.1
Source: Nat Commun. 2020 Jan 21;11:402. doi: 10.1038/s41467-019-13960-2 (PMC6972792; doi:10.1038/s41467-019-13960-2)
Supplement: Supplementary file 7 — Source data [file 41467_2019_13960_MOESM7_ESM.zip › Source_Data/FigureS2/FigureS2B_MotifScanOutput/KmeansCluster8/homerResults/motif27.similar.html]

motif27

## Information for motif27

A
C
G
T
A
G
T
C
G
A
C
T
G
A
T
C
T
C
G
A
C
G
A
T
C
T
A
G
C
T
G
A
T
A
C
G
T
C
G
A
C
G
T
A
A
T
G
C
C
A
G
T
T
A
G
C
  
Reverse Opposite:  

A
T
C
G
G
T
C
A
T
A
C
G
G
C
A
T
A
G
C
T
A
T
G
C
G
A
C
T
G
A
T
C
G
C
T
A
A
G
C
T
C
T
A
G
C
G
T
A
T
C
A
G
G
T
C
A
  

|  |  |
| --- | --- |
| p-value: | 1e-19 |
| log p-value: | -4.428e+01 |
| Information Content per bp: | 1.641 |
| Number of Target Sequences with motif | 58.0 |
| Percentage of Target Sequences with motif | 3.84% |
| Number of Background Sequences with motif | 375.5 |
| Percentage of Background Sequences with motif | 0.83% |
| Average Position of motif in Targets | 105.8 +/- 45.1bp |
| Average Position of motif in Background | 99.4 +/- 80.4bp |
| Strand Bias (log2 ratio + to - strand density) | 0.2 |
| Multiplicity (# of sites on avg that occur together) | 1.00 |
| Motif File: | file (matrix) reverse opposite |

### Similar de novo motifs found

|  |  |  |  |  |  |  |  |
| --- | --- | --- | --- | --- | --- | --- | --- |
| Rank | Match Score | Redundant Motif | P-value | log P-value | % of Targets | % of Background | Motif file |
| 1 | 0.912 | A C G T A T G C A G C T G A T C C T G A G A C T T C A G T C G A T A C G C G T A C G A T A T G C G A C T | 1e-18 | -42.865427 | 2.78% | 0.43% | motif file (matrix) |
| 2 | 0.870 | T A C G G C A T C A G T T A G C G A C T A G T C T C G A G A C T T C A G C G T A A C T G T G C A | 1e-17 | -40.153982 | 3.31% | 0.68% | motif file (matrix) |
| 3 | 0.783 | T C A G G C T A A C G T A G T C A G C T G A T C C T G A G C A T C T A G C G T A C A T G | 1e-15 | -34.576766 | 3.57% | 0.92% | motif file (matrix) |
